# Structural Diversity of Photosystem I and Its Light-Harvesting System in Eukaryotic Algae and Plants

**DOI:** 10.3389/fpls.2021.781035

**Published:** 2021-11-30

**Authors:** Tianyu Bai, Lin Guo, Mingyu Xu, Lirong Tian

**Affiliations:** Ministry of Education Key Laboratory of Molecular and Cellular Biology, Hebei Collaboration Innovation Center for Cell Signaling and Environmental Adaptation, Hebei Key Laboratory of Molecular and Cellular Biology, College of Life Sciences, Hebei Normal University, Shijiazhuang, China

**Keywords:** eukaryotic algae, light-harvesting complex I, photosystem I, structure, plant, evolution

## Abstract

Photosystem I (PSI) is one of the most efficient photoelectric apparatus in nature, converting solar energy into condensed chemical energy with almost 100% quantum efficiency. The ability of PSI to attain such high conversion efficiency depends on the precise spatial arrangement of its protein subunits and binding cofactors. The PSI structures of oxygenic photosynthetic organisms, namely cyanobacteria, eukaryotic algae, and plants, have undergone great variation during their evolution, especially in eukaryotic algae and vascular plants for which light-harvesting complexes (LHCI) developed that surround the PSI core complex. A detailed understanding of the functional and structural properties of this PSI-LHCI is not only an important foundation for understanding the evolution of photosynthetic organisms but is also useful for designing future artificial photochemical devices. Recently, the structures of such PSI-LHCI supercomplexes from red alga, green alga, diatoms, and plants were determined by X-ray crystallography and single-particle cryo-electron microscopy (cryo-EM). These findings provide new insights into the various structural adjustments of PSI, especially with respect to the diversity of peripheral antenna systems arising *via* evolutionary processes. Here, we review the structural details of the PSI tetramer in cyanobacteria and the PSI-LHCI and PSI-LHCI-LHCII supercomplexes from different algae and plants, and then discuss the diversity of PSI-LHCI in oxygenic photosynthesis organisms.

## Introduction

Oxygenic photosynthesis converts solar energy into biologically useful chemical energy and provides all life forms with oxygen, food, and fuel. Oxygenic photosynthetic organisms include cyanobacteria, eukaryotic algae, and plants; they all possess photosystem I (PSI) and photosystem II (PSII), which are embedded in thylakoid membranes and responsible for light-driven electron transport. Both PSI and PSII are multi-subunit pigment-protein supercomplexes consisting of the core complex and a light-harvesting complex (LHCI for PSI and LHCII for PSII, respectively) in eukaryotic algae and plants. PSII mediates the electrons’ transfer from water to the plastoquinone pool and produces electrons, protons, and molecular oxygen, whereas PSI mediates electron transfer from plastocyanin to ferredoxin and generates the power to reduce NADP^+^ into NADPH, which is eventually utilized to produce organic matter. PSI is among the most efficient nano-photochemical machines in nature: it converts solar energy into condensed chemical energy at nearly 100% quantum efficiency (Nelson, 2009; Croce and van Amerongen, 2013); hence, every captured photon by PSI can be used for electron translocation. Its conversion efficiency depends on the precise spatial arrangement of protein subunits and the extremely high content of non-protein components. In plant, each PSI-LHCI super-complex contains 16 subunits, 45 transmembrane helices, and more than 200 cofactors, including chlorophylls, carotenoids, phyloqunones, Fe_4_S_4_ clusters, lipids, and water molecules (Mazor et al., 2015, 2017; Qin et al., 2015; Wang et al., 2021).

Oxygenic photosynthesis evolved in primordial cyanobacteria ca. 2.5 billion years ago, from which eukaryotic photosynthetic cells developed through endosymbiosis, resulting in the evolvement of algae and plants (Dismukes et al., 2001; Nozaki, 2005). According to this endosymbiosis hypothesis, eukaryotic photosynthetic organisms incorporated an ancient cyanobacterium *via* primary endosymbiosis, leading to the formation of chloroplasts. These photosynthetic eukaryotes later divided into three lineages according to their color: chlorophytes (land plants plus green algae), rhodophytes (red algae), and glaucophytes. Red algae and those algae with secondary plastids of red algal origin are known as the “red lineage,” distinguishable from the “green lineage” (i.e., chlorophytes and organisms with secondary plastids of chlorophyte origin) (Sturm et al., 2013). Despite the long-term evolutionary interval between primordial cyanobacteria and plants, PSI has maintained its fundamental mechanism of sunlight conversion. The core complexes of PSI, from cyanobacteria to vascular plants, are highly conserved, whereas its peripheral antenna (LHCI) varies substantially in eukaryotic algae and vascular plants because of adaptations to different ecological niches. Oxyphototrophs in the green and red lineages harbor different types of pigments bound to their LHCs: in green lineage they bind chlorophyll *a*/*b*, whereas in the red algae they bind chlorophyll *a* termed Lhcr, and in the diatoms and brown algae they bind unique Chl *a*/*c* and fucoxanthin termed FCP (Nagao et al., 2020). Both lineages also differ in their carotenoid composition (Pan et al., 2020). Collectively, these differences are important for oxyphototrophs’ survival under different light conditions (Suga and Shen, 2020).

Recent advances in single-particle cryo-electron microscopy (cryo-EM), X-ray free electron laser (XFEL), X-ray diffraction (XRD), and other techniques have revealed unprecedented structural and catalytic details of how photosynthetic electron transfer occurs in cyanobacterial PSI and PSII cores (Umena et al., 2011; Suga et al., 2015, 2017; Malavath et al., 2018; Kato et al., 2019; Zheng et al., 2019; Chen et al., 2020), as well as in PSI-LHCI and PSII-LHCII supercomplexes from different algae and plant species (Su et al., 2017, 2019; Pi et al., 2018; Qin et al., 2019; Suga et al., 2019; Nagao et al., 2020; Xu et al., 2020). In particular, single-particle cryo-EM plays a unique role in analyzing supermolecular complexes of larger molecular size in algae and plants. For example, PSI-LHCIs from red alga (Antoshvili et al., 2018; Pi et al., 2018), green alga (Qin et al., 2019; Su et al., 2019; Suga et al., 2019; Perez-Boerema et al., 2020), diatom (Nagao et al., 2020; Xu et al., 2020), and moss (Yan et al., 2021); PSII-LHCIIs with disparate molecular weights; and special energy balancing mechanisms, such as state transitions when forming PSI-LHCI-LHCII, have been all resolved by single-particle cryo-EM. We next focus on recent research advances in PSI oligomers of cyanobacteria, the detailed structure of PSI-LHCI and PSI-LHCI-LHCII supercomplexes from algae and plants, and discuss the evolutionary process of PSI-LHCI in oxygenic photosynthesis organisms.

### Oligomeric State and Antenna System of Photosystem I in Cyanobacteria and Eukaryotic Organisms

**FIGURE 1:**
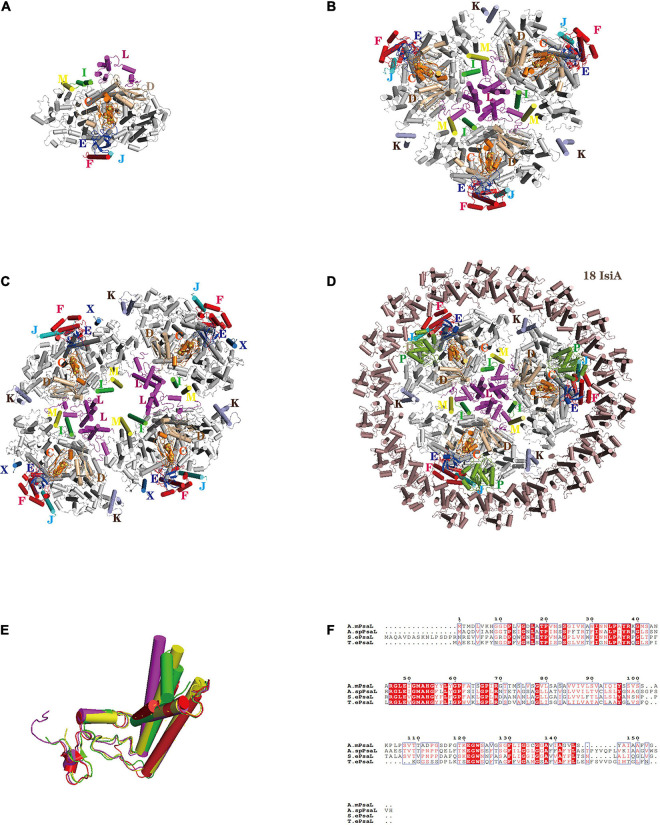
Structures of PSI oligomers from cyanobacteria. **(A)** PSI core monomer from *Thermosynechococcus elongatus* (6LU1). **(B)** Cyanobacterial PSI core trimer (7COY). **(C)** PSI core tetramer from *Anabaena* sp. PCC 7120 (6JEO). **(D)** PSI-IsiA supercomplex from *Synechococcus elongatus* PCC 7942 (6KIF). **(E)** Superposition of PsaL from the PSI core monomer in *Thermosynechococcus elongatus* (6LU1), the PSI core trimer (7COY), the PSI core tetramer in *Anabaena* sp. PCC 7120 (6JEO), and the PSI-IsiA supercomplex *Synechococcus elongatus* PCC 7942 (6KIF). **(F)** Sequence alignment of the PsaL protein from the PSI core monomer, PSI core trimer, PSI core tetramer, and PSI-IsiA supercomplex. The abbreviations used are as follows: A. m.: *Acaryochloris marina* (cyanobacteria), S. e.: *Synechococcus elongatus* PCC 7942 (cyanobacteria), T. e.: *Thermosynechococcus elongatus* (cyanobacteria), A. sp.: *Anabaena* sp. PCC 7120 (cyanobacteria). Color codes used are as follows (except PsaA and PsaB in panels **(A–D)** that are not labeled): PsaF, red; PsaI, green; PsaJ, cyan; PsaK, light-blue; PsaL, magenta; PsaM, yellow; PsaE, blue; PsaD, wheat; PsaP, split-pea. Color codes of PsaL in panel **(E)**: PsaL from *Thermosynechococcus elongatus* (red); PsaL from *Acaryochloris marina* (yellow); PsaL from *Anabaena* sp. PCC 7120 (magenta); PsaL from *Synechococcus elongatus* PCC 7942 (green).

The PSI in cyanobacteria is monomeric, trimeric or tetrameric, although it mainly exists as trimers *in vivo*. Almost 20 years ago, the structure of the prokaryotic PSI trimer from the cyanobacterium *Thermosynechococcus elongatus* was resolved at a high-resolution of 2.5 Å by X-ray crystallography (Fromme et al., 2001; Jordan et al., 2001), a great breakthrough in revealing the detailed molecular organization of PSI. Recently, more prokaryotic PSI structures with special functions from a variety of cyanobacterium species and physiological conditions were resolved by single particle cryo-EM analyses (Figure 1): functional monomeric PSI from *T. elongatus* (Netzer-El et al., 2018; Çoruh et al., 2021), tetrameric PSI from the heterocyst-forming cyanobacteria *Anabaena* sp. PCC 7120 (Kato et al., 2019; Zheng et al., 2019), the unique PSI reaction center that binds chlorophyll *d* in *Acaryochloris marina* (Hamaguchi et al., 2021; Xu et al., 2021) and the most red-shifted chlorophyll *f* in *Halomicronema hongdechloris* (Kato et al., 2020). The physiological functioning and evolutionary significance of the PSI tetramer prevalent in heterocyst-forming cyanobacteria has garnered much attention (Li et al., 2014, 2019; Semchonok et al., 2016). Structural analysis has uncovered unique monomer–monomer interactions in the PSI tetramer. Further, in cyanobacteria, the N-terminal, C-terminal and a middle region of PsaL each show marked variation between the trimer and tetramer of PSI, suggesting the spatial structure change of PsaL may have led to the tetramer formation (Figures 1E,F; Kato et al., 2019; Zheng et al., 2019). That formation might have been crucial for cyanobacteria to adapt to intense radiation, because a tetramer structure can increase the content of PSI-bound carotenoids, which may play a photoprotective role under high light incidence (Li et al., 2019). That study also supports the view that tetrameric PSI may be an intermediate in the evolution of cyanobacteria’s trimeric PSI to the monomeric PSI of plants and algae.

In cyanobacteria, each PSI core binds 12 protein subunits (from PsaA to PsaF, PsaI to PsaM, PsaX) to which 127 cofactors are non-covalently bound. Normally, cyanobacteria’s PSI lacks the peripheral antenna system of eukaryotic algae, but in some cases the hydrophilic phycobilidomes can be used for additional light-trapping antennas (Liu et al., 2013; Watanabe et al., 2014; Chang et al., 2015). Interestingly, under stressful conditions—especially in low-iron environments—cyanobacterial PSI express much iron stress-induced protein A (IsiA) that increase PSI’s effective cross-sectional absorption (Bibby et al., 2001; Boekema et al., 2001; Michel and Pistorius, 2004; Yeremenko et al., 2004). Like the PSII intrinsic antenna subunit CP43, the IsiA monomer has six transmembrane helixes and coordinates 17 chlorophyll molecules (Toporik et al., 2019; Cao et al., 2020). Electron microscopy studies showed that under iron starvation one or two IsiA rings encircled a trimeric PSI core to form a PSI-IsiA supercomplex. The cryo-EM structures of the PSI-IsiA supercomplex from the mesophilic cyanobacterium *Synechocystis* sp. PCC 6803 and *Synechococcus* sp. PCC 7942 showed that each PSI trimer is surrounded by 18 IsiA subunits, forming a closed ring (Figure 1D; Boekema et al., 2001; Toporik et al., 2019; Cao et al., 2020). The largest PSI-IsiA supercomplex consists of a PSI core trimer and 43 IsiA monomers having a double-ring shape (Yeremenko et al., 2004).

Unlike cyanobacteria’s PSI trimer or tetramer, eukaryotic PSIs in algae and plants are stably associated with LHC antenna subunits to form PSI-LHCI monomers. In eukaryotes, the crystal structure of PSI-LHCI in plants was first resolved at a 4.4-Å resolution from *Pisum sativum* (Ben-Shem et al., 2003), a monumental breakthrough for examining vascular plants’ PSI structure; this resolution has since been enhanced to 2.4 Å by X-ray crystallography (Mazor et al., 2015, 2017; Qin et al., 2015; Wang et al., 2021). In plants, each PSI core usually binds four LHCIs consisting of two separate dimers (i.e., proteins encoded by *lhca1*/*lhca4* and *lhca2*/ *lhca3*) (Wientjes and Croce, 2011), which together form a semispherical belt attached to the PsaF/PsaJ side. Although plants reportedly have six *Lhca* genes (*lhca1–6*), both *lhca5* and *lhca6* are only expressed at sub-stoichiometric levels (Pan et al., 2020). Unlike the PSI-LHCI in vascular plants, the number of PSI peripheral antennas in eukaryotic algae varies considerably. More than one PSI-LHCI structure may exist among the algae (e.g., Busch and Hippler, 2011; Drop et al., 2011). Recently, the PSI-LHCI structure from a red alga (*Cyanidioschyzon merolae*) and a green alga (*Chlamydomonas reinhardtii*) provided strong evidence that, indeed, algae do harbor more abundant forms of PSI-LHCI structures. In *C. merolae*, a typical thermophilic, acidophilic red alga, each PSI core binds 3 or 5 LHCRs (Antoshvili et al., 2018; Pi et al., 2018), whereas in *C. reinhardtii* each PSI core can bind 8 or 10 LHCIs (Qin et al., 2019; Su et al., 2019; Suga et al., 2019). These studies further emphasize the great structural variability of PSI-LHCI in eukaryotic algae.

### The Structure of PSI-LHCR in the Red Alga

**FIGURE 2:**
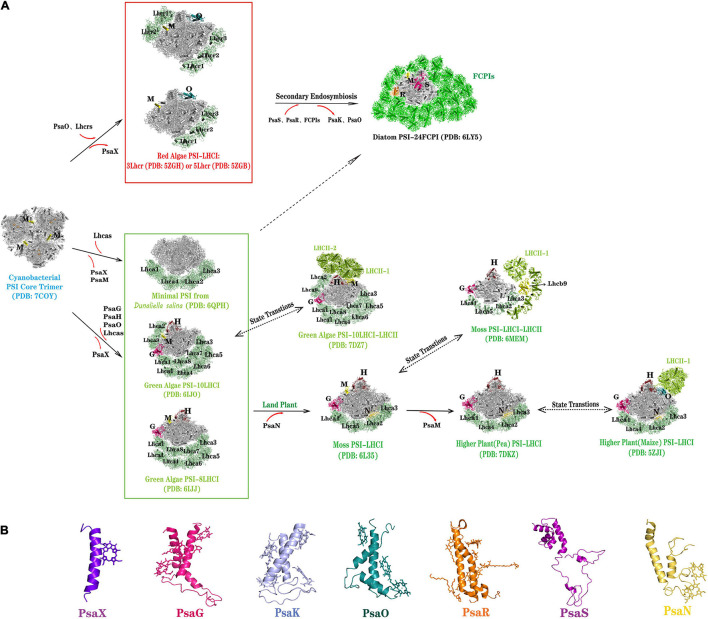
Diversity of PSI-LHCI supercomplexes and PSI core subunits deriving from oxygenic photosynthesis organisms. **(A)** Different PSI-LHCI from oxygenic photosynthesis organisms. Light-harvesting antenna Lhcrs and the core subunit PsaO are appeared in red algae. In the second endosymbiosis event, the core subunits PsaS, PsaR and light-harvesting antenna FCPIs are appeared in diatoms, whereas PsaK and PsaO are not found in diatoms. The light-harvesting antenna Lhcas (8 or 10) and the core subunits PsaG, PsaH, and PsaO are appeared in green algae (*C. reinhardtii* and *B. corticulans*), except for the mini-PSI from *Dunaliella salina* which contains seven core subunits and four Lhcas. PsaX is lost in all photosynthetic eukaryotes. The peripheral light-harvesting antenna of PSI is simplified in bryophytes and vascular plants, and the core subunit PsaM of PSI is lost, but PsaN is appeared in plant PSI. LHCII binds to the PSI-LHCI supercomplex when the state transition occurs in green algae, bryophytes, and vascular plants. **(B)** Structure of the special PSI core subunits from different photosynthetic organisms: transmembrane core subunits—PsaX, PsaG, PsaK, PsaO, PsaR, and PsaN, and non-transmembrane core subunit —PsaS. Color codes used are as follows (PsaA, PsaB, PsaC, PsaD, PsaE, PsaF, PsaI, and PsaK are uniformly represented in gray because they are relatively conserved in the positions of different species): PsaX, purpleblue; PsaG, hot pink; PsaH, ruby; PsaK, light blue in panel **(B)**; PsaM, yellow; PsaN, yellow-orange; PsaO, deep teal; PsaS, purple; PsaR, orange; FCPIs, green; LHCII, limon; lhcr/lhca, palegree.

Under environmental selective pressure, eukaryotic algae gradually evolved *via* phagocytosis, and their PSI also evolved a peripheral antenna system with a stronger light-harvesting ability during the evolutionary process. In the red lineage, algae (Rhodophyta) have similar characteristics to cyanobacteria, namely an absence of flagella and the presence of phycobiliproteins in their plastids (chloroplasts) (Nozaki, 2005); thus, red algae are considered primitive and related to cyanobacteria. Consequently, PSI core subunits in red algae and cyanobacteria are mostly conserved. Nevertheless, compared with cyanobacteria’s PSI, red algal PSI evolved a PsaO subunit at the PsaL/A/K side (Tian et al., 2017), and lost the PsaX. Moreover, red algae evolved chlorophyll *a*-binding proteins, these solely associated with PSI (Figure 2; Wolfe et al., 1994a, b). Compared to cyanobacteria and vascular plants, red alga has the shortest PsaL structure, whose N-terminal region lacks the three small β-strands connecting PsaL to the core in cyanobacteria and vascular plants (Antoshvili et al., 2018; Pi et al., 2018), while the C-terminal region lacks the short α-helix contributing to cyanobacterial PSI trimer’s formation. This simplified PsaL structure and novel subunits PsaO and Lhcrs attached to PSI core surfaces may explain the formation of a monomeric PSI-LHCR supercomplex. The Lhcrs’ arrangement in red algae is very interesting, occurring on both sides of the PSI core in the PSI-LHCR structure from *C. merolae* (Pi et al., 2018). Evidently, this arrangement is better for capturing incoming light energy from different directions of the thylakoid membrane and for effectively transmitting it to the reaction center.

### The Structures of PSI-LHCI and PSI-LHCI-LHCII in the Green Lineage

Oxyphototrophs in the green lineage mainly include green algae, mosses, and land plants. To date, PSI-LHCI’s structures—from three green algae (*C. reinhardtii, Bryopsis corticulans*, and *Dunaliella salina*), a moss (*Physcomitrella patens*) and two terrestrial plants (*P. sativum* and *Zea mays*)—has been determined, providing valuable insights into the evolution of PSI in this lineage (Ben-Shem et al., 2003; Qin et al., 2015, 2019; Su et al., 2019; Suga et al., 2019; Pan et al., 2020; Perez-Boerema et al., 2020; Wang et al., 2021; Yan et al., 2021). For the green algae, the structures of PSI-LHCI in *B. corticulans* living in marine water and *C. reinhardtii* living in freshwater are very similar, with only a slight difference: each PSI core binds 10 LHCI in *B. corticulans* whereas each PSI core can bind 8 or 10 LHCI in *C. reinhardtii*. Surprisingly, a simplified PSI-LHCI structure has been discovered from salt-tolerant *D. salina*, which contains only seven core subunits (PsaA–F and PsaJ) and four LHC proteins (Lhca1–4). Compared plants and green alga (*C. reinhardtii* and *B. corticulans*) binding with 14–16 subunits, *D. salina* forms the smallest known PSI-LHCI and may represent an acclimation response to hypersaline environments (Perez-Boerema et al., 2020).

Compared with the PSI core of cyanobacteria and red lineage organisms, green lineage oxyphototrophs evolved two significant membrane subunits: PsaG and PsaH (Figure 2B). The PsaG is homologous to PsaK and situated opposite of PsaK. The PSI-LHCI structure in plants and mosses suggests PsaG serves as an anchor for binding one Lhca at the first Lhca position. PsaH is located at the PsaL pole of the core complex and contains a single transmembrane helix and an additional helix encircling the PsaL subunit. Green lineage oxyphototrophs have retained their PSI monomer configuration albeit governed by the newly emerged PsaH subunit. The plant PSI features an extrinsic 10-kDa subunit PsaN located on the lumenal side close to PsaF, involved in the docking of plastocyanin (Amunts et al., 2007). Green lineage oxyphototrophs lack the PsaX of cyanobacteria and did not evolve the PsaR and PsaS seen in diatoms (Figure 2B).

A key feature of PSIs in green lineage oxyphototrophs is the differing amount of chlorophyll *a/b* proteins binding around the PSI core. Notably, the LHCI is about 2–3 times larger in unicellular green algae than vascular plants (Germano et al., 2002; Kargul et al., 2003). The structures of PSI-LHCI supercomplexes from *C. reinhardtii* suggest each PSI core can bind 8 or 10 LHCIs (Su et al., 2019; Suga et al., 2019). Eight LHCIs bind at the PsaF/PsaJ side, arranged into two layers as parallel half-rings. Each layer contains four LHCI proteins arranged in a crescent shape, forming the inner and outer LHCI belt. The additional two LHCIs are located near PsaB, at a similar position to those in the red algal PSI-LHCR. Interestingly, extra peripheral antennas are found at the PSI core’s PsaB side in both red and green algae. Hence, LHCIs tend to be arranged on different sides of the PSI core in eukaryotic algae during the primary endosymbiosis evolution, likely critical for their adaption to shifting light intensities in aquatic environments.

In the green lineage, bryophytes (i.e., liverworts, mosses, hornworts) diverged from the ancestor of seed plants and the structure and function of their PSI-LHCI represents PSI’s evolution from aquatic to terrestrial life (Falkowski, 2006; Iwai and Yokono, 2017). The PSI-LHCI supercomplex from the moss *P. patens* was structurally similar to land plants’ PSI-LHCI, except Lhca5 in the former instead of Lhca4 in the latter (Busch et al., 2013; Yan et al., 2021). Yet the *P. patens* genome codes for more diverse and redundant light-harvesting antenna proteins than those of green algae and land plants, suggesting complicated PSI-LHCI structures exist in *P. patens* (Busch et al., 2013; Iwai and Yokono, 2017). The PSI-LHCI from vascular plants—determined by X-ray crystallography at higher resolutions—is composed of 19 protein subunits and ca. 200 non-covalently bound cofactors (Ben-Shem et al., 2003). Hence, structure of PSI-LHCI gradually stabilized during its aquatic-to-terrestrial evolutionary transition.

Interestingly, in the green lineage, exists a short-term light-adaptation process to regulate the light-harvesting capacity of PSI and PSII, this termed “state transition”. During this process, a portion of LHCII is phosphorylated and dissociated from PSII and moved to PSI, forming a PSI-LHCI-LHCII supercomplex. To date, the molecular structure of PSI-LHCI-LHCII supercomplex has been reported for green algae, mosses, and land plants (Figure 2A; Iwai et al., 2015, 2018; Pan et al., 2018; Pinnola et al., 2018; Huang et al., 2021). The PSI-LHCI-LHCII supercomplex from *C. reinhardtii* has been determined to a 3.42 and 2.84-Å resolution by cryo-EM (Huang et al., 2021; Pan et al., 2021). Examination of the *Cr*PSI-LHCI-LHCII supercomplex structure showed that each PSI core binds 10 LHCI proteins and two LHCII trimers, the latter forming an additional antenna belt at the PsaH/L/O side. In *P. patens*, a newly LHCII protein-Lhcb9 associated with PSI was found, forming a large PSI-LHCI-LHCII supramolecular complex, in which each PSI core may bind two rows of LHCI belt, one copy of Lhcb9, and a LHCII trimer (Iwai et al., 2018; Pinnola et al., 2018). The PSI-LHCI-LHCII supercomplex in plants (*Z. mays*) is smaller than that identified from *C. reinhardtii* and *P. patens* and it contains only one LHCI belt (Lhca1-Lhca4) and one LHCII trimer. The LHCII trimer in plants is located at a position similar to that in the PSI-LHCI-LHCII supercomplex of green alga and moss. However, the other LHCII trimer in *Cr*PSI-LHCI-LHCII is specific to green alga and bridges the gap between the conserved LHCII trimer and Lhca2. In summary, compared with PSI of green algae and mosses, which has diverse structure of the peripheral light-harvesting complex, the plants’ PSI-LHCI and PSI-LHCI-LHCI evolved to become more stable, likely because vascular plants face stronger light exposure conditions after moving from an aqueous to terrestrial environment.

### The Structure of PSI-FCPI in the Diatoms

Diatoms and brown algae are derived from a secondary endosymbiotic event, and their chloroplasts are surrounded by four membranes (Veith et al., 2009). Recently, the organization and structure of the PSI-FCPI (fucoxanthin Chl *a*/*c*-binding proteins) supercomplex from the diatom *Chaetoceros gracilis* was reported at a 2.38 and 2.40-Å resolution, respectively, by cryo-EM (Nagao et al., 2020; Xu et al., 2020). These findings confirmed the PSI’s organization in red-lineage oxyphototrophs through a secondary endosymbiosis event. Compared that of PSI-LHCR in red algae, the structure of PSI in diatoms evolved two new subunits, PsaR and PsaS, but lost PsaK and PsaO (Figure 2B). PsaR is a unique membrane-spanning subunit phylogenetically close to PsaG in green lineage organisms, but it has a low homology with PsaG. Nevertheless, PsaR’s role may be similar to PsaG’s in these organisms: mediating the binding and energy transfer of FCPI to the PSI core. The diatom PSI core lost the PsaK that exists in all oxyphototrophs and PsaO typical of eukaryotic organisms, which may be related to the unique secondary endosymbiosis position of diatoms. The most striking difference between the red algal and diatom PSIs is the monomeric diatom PSI core being surrounded by 24 FCPI antennas (Nagao et al., 2020; Xu et al., 2020), amounting to 1.1 MDa (molecular weight), the largest known monomeric PSI-LHCI supercomplex of any photosynthetic organisms. Special fucoxanthin (Fx) chlorophyll *a*/*c* antenna proteins in diatoms can help to efficiently harvest blue-green light under water (400–550 nm), which is essential for their survival and fitness in aquatic environments (Wang et al., 2020).

## Conclusion and Perspectives

Red and green lineages constitute the main evolutionary lines of eukaryotic photosynthetic organisms that arose from a common ancestor through an endosymbiotic event, whereby a cyanobacterium ancestor gave origin to the eukaryotic chloroplast. Both the PSI core complex and its peripheral antennae in photosynthesis organisms exhibit significant structure diversity in terms of oligomerization state, core subunit composition, subunit number and composition of the LHCI antenna. These are important for photosynthetic organisms to adapt to various environments. Although much structural information about PSI-LHCI has been gleaned by advanced biophysical techniques (e.g., cryo-EM), we still know little of dynamic interaction processes in the thylakoid membrane—such as cyclic electron flow and binding of photoprotective proteins to photosystems—involved in light-acclimation responses. In particular, the recent discovery of the PSI-LHCI homodimer from *C. reinhardtii* further indicates the structural plasticity and diversity of PSI (Naschberger et al., 2021). Whether the macromolecular organization such as the PSI-LHCI dimer has a physiological role in the maintenance of chloroplast thylakoid membrane structure needs to be deeply studied.

## Author Contributions

TB and LG wrote the manuscript with the help of MX. LT reviewed and revised the manuscript. All authors contributed to the article and approved the submitted version.

## Conflict of Interest

The authors declare that the research was conducted in the absence of any commercial or financial relationships that could be construed as a potential conflict of interest.

## Publisher’s Note

All claims expressed in this article are solely those of the authors and do not necessarily represent those of their affiliated organizations, or those of the publisher, the editors and the reviewers. Any product that may be evaluated in this article, or claim that may be made by its manufacturer, is not guaranteed or endorsed by the publisher.
